# Mechanistic insight of *Staphylococcus aureus* associated skin cancer in humans by *Santalum album* derived phytochemicals: an extensive computational and experimental approaches

**DOI:** 10.3389/fchem.2023.1273408

**Published:** 2023-11-21

**Authors:** Md. Eram Hosen, Sumaiya Jahan Supti, Shopnil Akash, Md. Ekhtiar Rahman, Md Omar Faruqe, M. Manirujjaman, Uzzal Kumar Acharjee, Abdel-Rhman Z. Gaafar, Lahcen Ouahmane, Baye Sitotaw, Mohammed Bourhia, Rashed Zaman

**Affiliations:** ^1^ Professor Joarder DNA and Chromosome Research Laboratory, Department of Genetic Engineering and Biotechnology, University of Rajshahi, Rajshahi, Bangladesh; ^2^ Department of Genetic Engineering and Biotechnology, University of Rajshahi, Rajshahi, Bangladesh; ^3^ Department of Pharmacy, Faculty of Allied Health Sciences, Daffodil International University, Dhaka, Bangladesh; ^4^ Department of Computer Science and Engineering, University of Rajshahi, Rajshahi, Bangladesh; ^5^ Department of Structural and Cellular Biology, Tulane University School of Medicine, New Orleans, LA, United States; ^6^ Department of Genetics, University of Alabama, Birmingham, AL, United States; ^7^ Department of Botany and Microbiology, College of Science, King Saud University, Riyadh, Saudi Arabia; ^8^ Laboratory of Microbial Biotechnologies, Agrosciences and Environment (BioMAgE), Labeled Research Unit-CNRSTN°4, Cadi Ayyad University, Marrakesh, Morocco; ^9^ Department of Biology, Bahir Dar University, Bahir Dar, Ethiopia; ^10^ Department of Chemistry and Biochemistry, Faculty of Medicine and Pharmacy, Ibn Zohr University, Laayoune, Morocco

**Keywords:** skin cancer, *Staphylococcus aureus*, staphylococcal accessory regulator (SarA), *Santalum album*, molecular docking, molecular dynamics, ADMET prediction, antibacterial activity

## Abstract

An excessive amount of multidrug-resistant *Staphylococcus aureus* is commonly associated with actinic keratosis (AK) and squamous cell carcinoma (SCC) by secreted virulence products that induced the chronic inflammation leading to skin cancer which is regulated by staphylococcal accessory regulator (SarA). It is worth noting that there is currently no existing published study that reports on the inhibitory activity of phytochemicals derived from *Santalum album* on the SarA protein through *in silico* approach. Therefore, our study has been designed to find the potential inhibitors of *S. aureu*s SarA protein from *S. album*-derived phytochemicals. The molecular docking study was performed targeting the SarA protein of *S. aureus*, and CID:5280441, CID:162350, and CID: 5281675 compounds showed the highest binding energy with −9.4 kcal/mol, −9.0 kcal/mol, and −8.6 kcal/mol respectively. Further, molecular dynamics simulation revealed that the docked complexes were relatively stable during the 100 ns simulation period whereas the MMPBSA binding free energy proposed that the ligands were sustained with their binding site. All three complexes were found to be similar in distribution with the apoprotein through PCA analysis indicating conformational stability throughout the MD simulation. Moreover, all three compounds’ ADMET profiles revealed positive results, and the AMES test did not show any toxicity whereas the pharmacophore study also indicates a closer match between the pharmacophore model and the compounds. After comprehensive *in silico* studies we evolved three best compounds, namely, Vitexin, Isovitexin, and Orientin, which were conducted *in vitro* assay for further confirmation of their inhibitory activity and results exhibited all of these compounds showed strong inhibitory activity against *S. aureus.* The overall result suggests that these compounds could be used as a natural lead to inhibit the pathogenesis of *S. aureus* and antibiotic therapy for *S. aureus*-associated skin cancer in humans as well.

## Graphical Abstract

**Figure:**
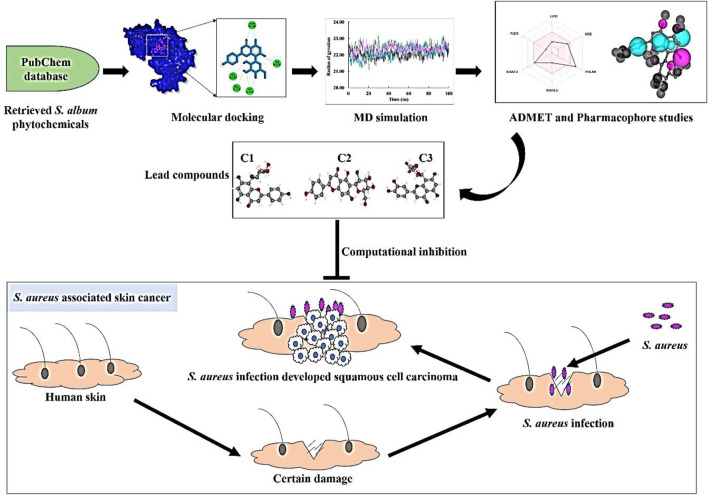

## 1 Introduction

Skin, the largest organ of the human body, is vulnerable to various diseases, with skin cancer being a significant concern (Zhang et al., 2020). Actinic keratosis (AK), a premalignant lesion, can lead to the development of squamous cell carcinoma (SCC), a common type of skin cancer (Krueger et al., 2022b). Chronic inflammation plays a crucial role in the progression from AK to SCC, particularly in individuals with inflammatory skin disorders. SCC tends to develop in areas of the skin that are chronically inflamed, such as burns, wounds, and ulcers (Ciążyńska et al., 2021). The chronic inflammation associated with skin cancer is often driven by the synthesis of reactive oxygen species (ROS), leading to DNA damage and genomic instability (Chakraborty et al., 2020). The skin is inhabited by various microorganisms, including bacteria, fungi, archaea, and viruses, collectively known as the skin microbiome. The microbiome has been found to influence skin inflammation. Certain microbes, such as *Staphylococcus aureus*, *Helicobacter pylori*, *Salmonella typhi*, *Escherichia coli*, and human *papillomavirus*, possess carcinogenic potential. These microorganisms secrete products that induce oxidative stress and DNA damage, contributing to skin cancer development (Byrd Allyson et al., 2018; Okunade, 2020; Krueger et al., 2022a).

*S. aureus* is a highly detrimental human pathogenic bacterium and a leading cause of healthcare-associated infections. It is estimated that *S. aureus* colonizes approximately 30% of the human population (Oliveira et al., 2022). According to the CDC, in 2011, there were 80,461 reported cases and 11,285 fatalities in the United States due to invasive *S. aureus* infections (Reimche et al., 2021). This gram-positive bacterium is commonly found on the skin and mucous membranes, and it is responsible for a wide range of infections, including bacteremia, infective endocarditis, skin and soft tissue infections, osteomyelitis, septic arthritis, pulmonary infections, gastroenteritis, meningitis and urinary tract infections (Taylor and Unakal, 2022). An excessive presence of multidrug-resistant *S. aureus* is often associated with the development of skin cancer. The colonization of *S. aureus* on precancerous skin and the secretion of virulence products contribute to the progression of skin cancer (Liu et al., 2018; Madhusudhan et al., 2020; Krueger et al., 2022b).

**FIGURE 1:**
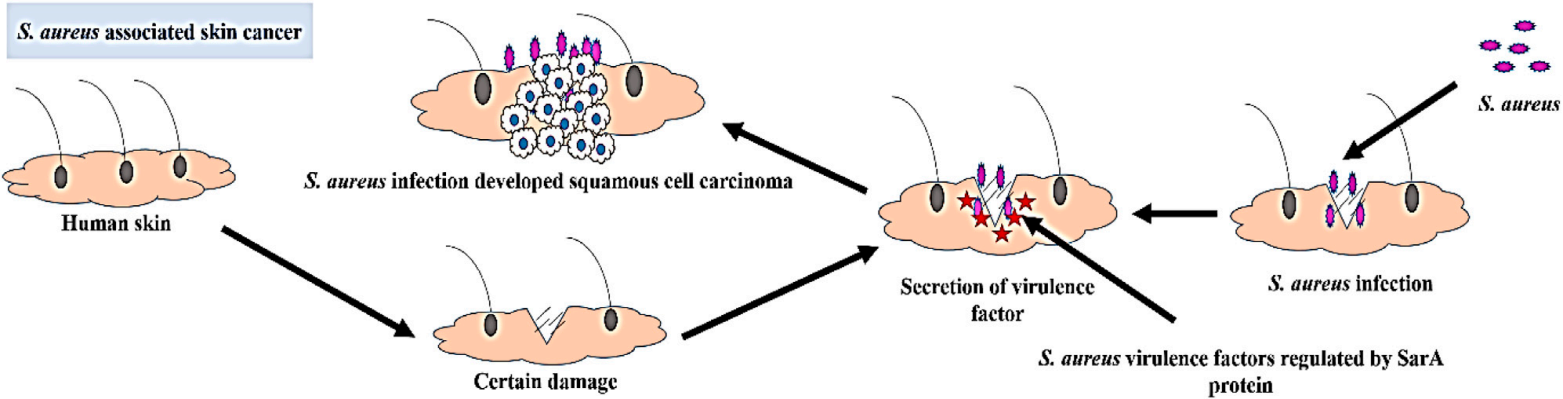
Schematic representation of the development of *S. aureus* associated skin cancer.

The occurrence of this widespread infection can be attributed to the remarkable diversity of extracellular and cell wall-associated virulence factors that are expressed in a coordinated manner during the infectious process (Wang et al., 2022). Many of these virulence components manifest as either secreted proteins or cell surface-associated proteins. Secreted proteins such as hemolysins, lipases, and proteolytic enzymes are responsible for invasion and tissue damage. On the other hand, adhesion to host tissues is mediated by cell surface-associated proteins, such as protein A and proteins that bind to fibronectin (El-Ganiny et al., 2022). The expression of these virulence factors is regulated by a key protein called staphylococcal accessory regulator (SarA), which plays a pivotal role in *S. aureus* pathogenesis (Wang et al., 2019). Upon certin damage in skin *S. aureus* get opportunity to infect where the staphylococcal accessory regulator (SarA) protein regulate the secretion of virulence factor which leads to the development of squamous cell carcinoma in that infection region (Figure 1). These virulence proteins induce chronic inflammation, which can lead to the development of skin cancer. SarA, a 124-residue (14.7-kDa) protein, binds to the promoter region of target genes and serves as a promising target for the development of antibiotic cancer therapy against *S. aureus*-associated skin cancer (Díaz et al., 2022).

The primary approach for treating *S. aureus* infections is the administration of antibiotic drugs from the *β*-lactam class, such as cephalosporins, oxacillin, or nafcillin (Blackman et al., 2020). However, in recent years, there has been increasing resistance of *S. aureus* to various antibiotic drugs, including methicillin, nafcillin, oxacillin, vancomycin, penicillin, cotrimoxazole, amoxicillin, tetracycline and cloxacillin (Shariati et al., 2020; Reimche et al., 2021). Although synthetic drugs remain the primary means of controlling these infections, they often come with significant side effects. Hence, there is a pressing need to explore alternative medicines that can effectively treat these bacteria while minimizing adverse effects. Therefore, SarA protein setected as a promising drug target to development of antibacterial drug for skin cancer associated bacteria *S. aureus.*

*S. album*, commonly known as sandalwood and belonging to the family Santalaceae, is a medicinal plant known for its rich phytochemical content and traditional use in treating a wide range of human diseases (Akbar and Akbar, 2020). In addition to its medicinal applications, sandalwood is also utilized in cosmetic products. The leaf extract of *S. album* exhibits diverse biological properties, including antimicrobial, antioxidant, and cytotoxic effects (Pullaiah et al., 2021). The leaves of this plant species are particularly abundant in alkaloids, flavonoids, terpenoids, tannins, phenolics, saponins, and steroids (Ghildiyal et al., 2020). These compounds contribute to the antimicrobial activity of *S. album*, making it a potential source for improving the development of antibacterial drugs.

This research aims to identify promising lead compounds that could inhibit the pathogenesis of *S. aureus* and serve as a potential therapy for *S. aureus*-associated skin cancer in humans.

## 2 Material and methods

### 2.1 Collection and preparation of *S. album* phytochemicals

**FIGURE 2:**
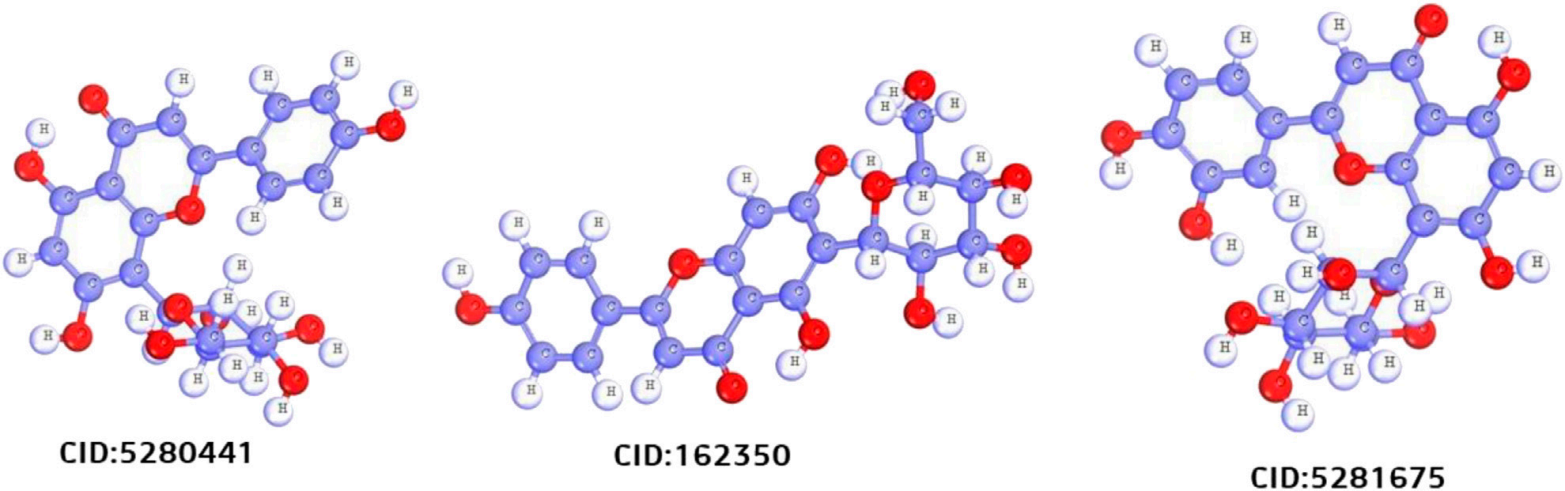
Scafold structure of three best compounds.

After a comprehensive literature review, 50 chemical compounds of *S. album* (Liu et al., 2008; Umdale et al., 2020) were retrieved in sdf format from the Pub-Chem database (Kim et al., 2021). The compounds were then cleaned, and energy was minimized using Avogadro software v1.2.0 with the help of mmf94 force field (Hanwell et al., 2012). The scaffold structure of the hit compounds shown in Figure 2.

### 2.2 Protein preparation

**FIGURE 3:**
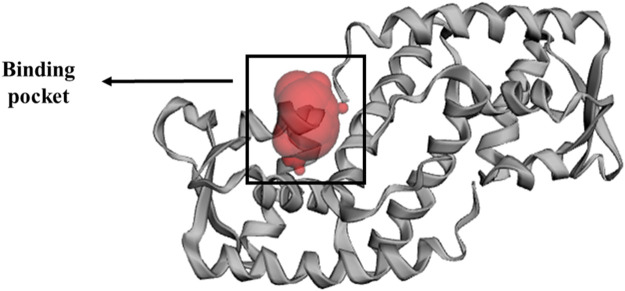
Binding pocket of SarA protein, identified by CASTp (http://sts.bioe.uic.edu/castp/index.html?2fnp) online server (Tian et al., 2018).

The x-ray crystallography structure of SarA protein of *S. aureus* (PDB ID: 2FNP) was retrieved from the protein data bank (Bouley et al., 2015)**.** With the aid of the software Discovery Studio v21.1.0.0 (https://discover.3ds.com/discovery-studio-visualizer-download), the protein structure was initially cleaned and heteroatoms were removed. Then, using the GROMOS96 43b1 force field and the SwissPDB Viewer software v4.1 (https://spdbv.unil.ch/disclaim.html) the energy of the cleaned protein was minimized and optimized (Guex and Peitsch, 1997). The active site and binding pocket of the target protein SarA (PDB: 2fnp) was identified by CASTp online server (Figure 3).

### 2.3 Molecular docking

The molecular docking between phytochemicals from *S. album* and SarA protein of *S. aureus* was carried out using PyRx software v0.8 (https://sourceforge.net/projects/pyrx/) which works based on the Auto Dock vina configuration (Dallakyan and Olson, 2015) and docking was performed based on the previous methods with small modification (Muhammad and Fatima, 2015; Bhowmik et al., 2021; Dey et al., 2023). The protein structure was converted into a macromolecule and the ligands were converted to PDBQT format. The center and grid box size of the docked complexes were set as for center X: 3.8347 Å, Y: −0.1554 Å, Z: 8.7332 Å and for dimension X: 48.7233 Å, Y: 41.3090 Å, Z: 59.7350 Å respectively. The final docking calculation was conducted using PyRx and top molecules were selected based on lower binding energy. The binding interactions and poses were explored via Discovery Studio software.

### 2.4 Molecular dynamics

YASARA (Yet Another Scientific Artificial Reality Application) Dynamics software v19.12.4 was used to conduct molecular dynamic simulation with the help of the Assisted Model Building with Energy Refinement (AMBER)14 force field (Supplementary Material S1) (Wang et al., 2004; Land and Humble, 2018). The hydrogen bond network was initially cleaned up and optimized together with the docked complexes. In order to reduce the protein complexes using a TIP3P water solvation model (0.997 g/L1, 25 c, 1 atm), the steepest gradient approaches were used (Harrach and Drossel, 2014). The physiological conditions were set at 0.9% NaCl, 310K, and pH 7.4 (Krieger et al., 2012) to neutralize the simulated system. The simulation time step was set as normal at 2 × 1.25 frames per second. The long-range electrostatic interaction was calculated by the particle mesh Ewald (PME) method with a cutoff radius of 8.0 Å (Essmann et al., 1995). The simulation trajectories were saved after every 100 ps and the final simulation run was conducted for 100 ns (Krieger and Vriend, 2015). The root-mean-square deviation, the solvent-accessible surface area, the radius of gyration, and hydrogen bonding were all assessed using the simulation trajectories (Baildya et al., 2021; Islam et al., 2021).

### 2.5 Binding-free energy calculation using MM/PBSA

Calculating the binding free energy is a crucial approach for evaluating the strength of the interaction between a drug and a protein. This analysis provides insights into the energetic aspects of the drug-protein complex. To determine the binding free energy, various snapshots of the complex were analyzed using the MM-Poisson–Boltzmann surface area (MM-PBSA) method in YASARA software. The calculation involved the following formula:

MM/PBSA binding free energy = EpotReceptor + EsolvReceptor + EpotLigand + EsolvLigand−EpotComplex−EsolvComplex.

The calculations were performed using the AMBER 14 force field, and YASARA macros were utilized for efficient computation of the MM-PBSA binding energy.

### 2.6 Principal components analysis

Principal component analysis (PCA) was utilized to explore the overall variability among protein-ligand complexes, including comparisons with the apo form and a drug-protein complex, by considering all the structural features (Akash et al., 2023b; 2023a). This method allows for the identification and categorization of structural changes in protein-ligand complexes that occur during the simulation by comparing different variables. Through diagonalization of the covariance matrices and solving the eigenvalue and eigenvector problems, PCA was performed on the complexes. The eigenvalues provided information about the magnitude and direction of structural fluctuations, while the eigenvectors represented the directions of these fluctuations. The MD trajectories spanning 100 ns were pre-processed and standardized by scaling to unit variance and removing the mean (Ichiye and Karplus, 1991; Shukla and Tripathi, 2020). The PCA technique was implemented using Python v3.11 with the Scikit-learn v1.2 library, and the results were visualized using Matplotlib v3.7 (Supplementary Material S2).

### 2.7 ADMET analysis

The phytochemicals that passed the docking study were then subjected to absorption, distribution, metabolism, excretion, and toxicity (ADMET) analysis to see whether they have the necessary qualities to be considered as lead molecules. Therefore, the pkcsm (Pires et al., 2015) and SwissADME (Daina et al., 2017) web servers were used to analyze ADMET profiles and calculate molecules’ adherence to Lipinski’s rule of five respectively.

### 2.8 Pharmacophore mapping

The pharmacophore mapping analysis of the top three ligands was performed using the online server PharmMapper (Wang et al., 2017). The ligands were obtained in sdf format from the PubChem server and then uploaded to the server. During the process, the “maximum number of conformations” parameter was set to 1,000. All available targets were selected in the “select target set” parameter, and the “number of reserved matched targets” parameter was set to 1,000. The fit score cut-off value in the advanced settings was set at 0. The default settings were used for all other parameters.

### 2.9 *In vitro* antibacterial activity

#### 2.9.1 Chemical and reagents

The compounds Vitexin (CID: 5280441), Isovitexin (CID: 162350), and Orientin (CID: 5281675) were purchased from Sigma-Aldrich as HPLC standards. Methanol, used for sample preparation, was of HPLC grade. The antibiotic drug ciprofloxacin was purchased from Square Pharmaceuticals Ltd. Luria Bertani (LB) broth and LB agar media were bought from Sigma-Aldrich (United States).

#### 2.9.2 Collection of bacterial sample

Skin cancer associated bacteria *S. aureus* were collected from Professor Joardar DNA and Chromosome Research Laboratory, Department of Genetic Engineering and Biotechnology, University of Rajshahi, Bangladesh. Which was previously clinically isolated from skin cancer patient. After collection, the bacteria were cultured in LB agar meida and allow to grow it at 37°C for overnight. For long-term storage *S. aureus* were kept at −80°C. *S. aureus* is classified as a Biosafety Level 2 (BSL-2) pathogen. Therefore, we follow all appropriate guidelines and regulations for the use and handling of this bacteria.

#### 2.9.3 Determination of *in vitro* antibacterial activity

The three most promising compounds, Vitexin (CID: 5280441), Isovitexin (CID: 162350), and Orientin (CID: 5281675), which exhibited significant inhibitory effects against *S. aureus* associated with skin cancer through *in silico* analysis, were chosen for further evaluation of their *in vitro* antibacterial activity against this skin cancer-associated bacteria. To prepare the test solutions, all three compounds were dissolved in 60% methanol and diluted to a concentration of 50 μg/mL. Subsequently, an *in vitro* antibacterial assessment was conducted using the disc diffusion method, with slight modifications (Tiruneh et al., 2022), at a concentration of 50 µg/disc. The bacterial cultures were initially grown overnight in nutrient broth at 37°C, with agitation at 180 rpm. Subsequently, an overnight bacterial suspension with a concentration of 1 × 10^6^ CFU/mL was evenly spread onto LB agar plates. Whatman No. 1 filter paper discs, each with a diameter of 5 mm, were utilized in the experiment. These discs were impregnated with 50 µg of each compound (Vitexin, Isovitexin, and Orientin) and thoughtfully positioned on the agar plates. Ciprofloxacin, an antibiotic drug, was employed as a positive control. After 24 h of incubation, the presence of clear zones around the discs indicated inhibition of bacterial growth. The diameters of these inhibition zones were measured using a millimeter (mm) scale. The experiment was repeated three times to ensure accuracy, and the data were subsequently presented as the mean and standard deviation of the results.

## 3 Results and discussion

### 3.1 Molecular docking study

*S. aureus* is associated with skin cancer by secreting virulence factors which are regulated by SarA protein (Bromfield et al., 2023). Interestingly, there was no published study has been reported on the inhibitory activity of *S. album* phytochemicals on SarA protein. Therefore, in this current investigation, we performed *in silico* docking study targeting SarA (PDB ID: 2fnp) protein of *S. aureus* which is responsible for the expression of many virulence genes (Jiang et al., 2023), by using *S. album* derived phytochemicals. The active site residue of SarA protein are A:Phe110, A:Ser114, A:Thr117, A: Thr118, A:Lys121, A:Glu223, A:Leu224, B:Thr141, B:Thr142, B:Glu145, B:Asn146, B:His159, and B:Tyr162 (Table 1). In our study, we found all three compounds strongly bind with the most of the active site residues of SarA protein including A:Phe110, A:Thr117, A:Lys121, A:Glu223, A:Leu224, B:Asn146, and B:His159 (Table 2), which indicates that these three compounds can potentially significant for the inhibition of the target protein SarA.

**TABLE 1:** Active side residue of SarA protein identified by CASTp online server (Tian et al., 2018).

Target protein	Active side residue
SarA (PDB: 2fnp)	A:Phe110, A:Ser114, A:Thr117, A: Thr118, A:Lys121, A:Glu223, A:Leu224, B:Thr141, B:Thr142, B:Glu145, B:Asn146, B:His159, B:Tyr162

**TABLE 2:** Non-covalent interaction of the ligand molecules against SarA protein of *Staphylococcus aureus*, binding energy, non-covalent interaction, interacting amino acids, bond types and their distance.

Complex	Binding energy (kcal/mole)	Amino acid residues	Bond types	Distance Å)	Angle (˚)
2fnp + CID:5280441	−9.4	B:Asn146	H	2.29	126.198
		A:Leu224	H	2.16	145.587
		A:Glu223	H	2.66	93.504
		A:Lys121	H	2.73	110.107
		B:His159	H	2.66	127.773
2fnp + CID:162350	−9.0	A:Glu221	H	2.06	136.664
		A:Glu223	H	2.24	161.549
		A:Lys121	H	2.77	123.346
		B:His159	H	2.40	134.646
		A:Phe110	PP	5.30	-
		B:Tyr142	PP	5.08	-
2fnp + CID:5281675	−8.6	B:Asn146	H	2.46	127.061
		B:Asn158	H	2.18	135.591
		A:Lys121	H	2.70	107.513
		B:His159	H	2.07	140.478
2fnp + CID:114776	−7.7	A:Lys121	H	2.94	122.446
		B:Asn146	H	2.41	134.582
		B:His159	H	2.33	131.92
2fnp + CID:5281654	−7.4	A:Glu223	H	2.78	102.168
		B:Asn146	H	2.17	150.899
		B:His159	H	2.44	138.895
2fnp + CID:2764 (Control)	−8.6	A:Thr117	H	2.38	107.41
		A:Asp120	H	3.28	105.05
		B:Tyr162	H	5.22	128.49
		B:Gln166	H	3.79	160.366

H, Hydrogen bond; PP, Pi-pi sigma bond.

In a molecular docking study, from 50 compounds of *S. album* top five compounds were chosen based on the lowest binding energy, interaction with target protein, pose and RMSD value. Compound CID: 5280441 found a maximum binding energy −9.4 kcal/mol followed by CID: 162350 and CID: 5281675 with binding energy of −9.0 kcal/mol and −8.6 kcal/mol respectively compared to the positive control ciprofloxacin CID: 2764 with −8.6 kcal/mol (Table 2).

**FIGURE 4:**
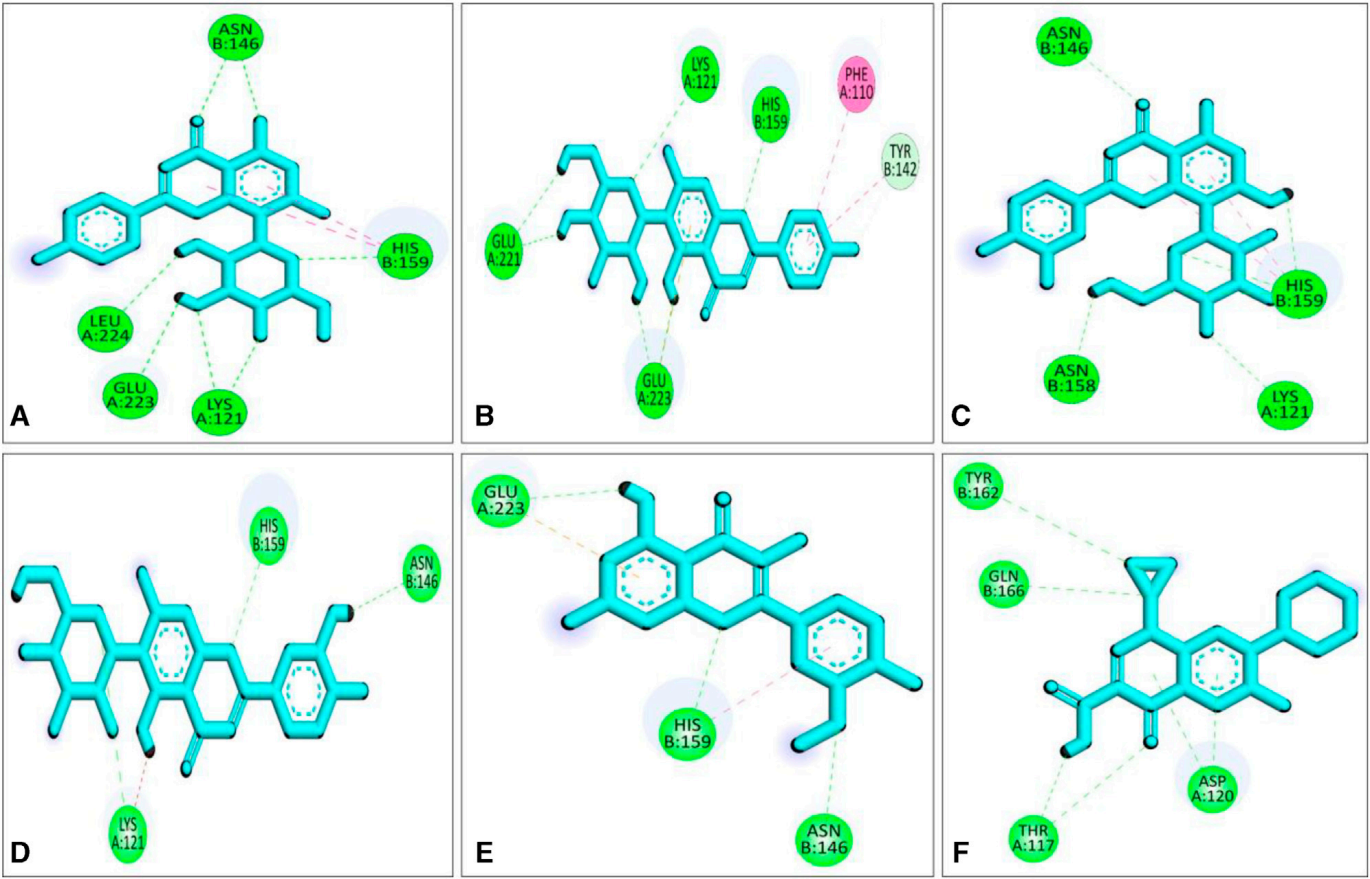
Molecular docking interactions of the compounds from *S. album* and SarA protein of *Staphylococcus aureus*; 2d view of compounds CID: 5280441 **(A)**, CID: 162350 **(B)**, CID: 5281675 **(C)**, CID: 114776 **(D)**, CID: 5281654 **(E)** and CID: 2764 **(F)** respectively. Figures were generated by using Discovery Studio v21.1.0.0.

Compared to the previous study our current compounds showed better binding affinity than compound hesperidin which interacts with active sites of SarA with the binding energy of −6.9 kcal/mol by revealing two hydrogen bonding interactions at Thr 117 and Lys 163 (Tong et al., 2015). Another study reported that benzimidazole type NHC precursors 1a-d molecules exhibited binding energy ranging from −5.04 to −5.46 kcal/mol with the SarA protein which is comparatively lower to our current findings and did not show any kinds of hydrogen bond (Üstün et al., 2021), the difference of the binding energy maybe due to presence of more hydrogen bond interaction between the protein and our current ligands (Figure 4). Some other studies also found that the SarA protein of *S. aureus* was inhibited by different compounds (de Oliveira et al., 2019; Cheruvanachari et al., 2023). However, in our study five hydrogen bonds were found from complex 2fnp + CID: 5280441 at B: Asn146 (2.29 Å), A:Leu224 (2.16Å), A:Glu223 (2.66Å), A:Lys121 (2.73Å) and B:His159 (2.66 Å) (Figure 4A). Similarly, Complex 2fnp + CID:162350 was stabilized by four hydrogen bonds at A:Glu221 (2.06 Å), A:Glu223 (2.24 Å), A:Lys121 (2.77Å) and B: His159 (2.40 Å) (Figure 4B). Moreover, the interaction between 2fnp and compounds CID: 5281675 revealed four hydrogen bonds in A chain of 2fnp protein at A:Lys121 (2.70 Å) and B chain of 2fnp protein at B: Asn146 (2.46 Å), B: Asn158 (2.18 Å) and B:His159 (2.07 Å) (Figure 4C). Additionally, the complexes 2fnp + CID: 114776 and 2fnp + CID: 5281654 showed almost similar binding energy with −7.7 and −7.4 kcal/mol respectively (Table 2), and also interact target protein with three hydrogen bonds where two residue such as B:Asn146 and B:His159 were same with different distance (Figures 4D, E).

In comparison with other three lead compounds, the positive control CID:2764 exhibited four hydrogen bond at A:Thr117 (2.38 Å), A:Asp120 (3.28 Å), B:Tyr162 (5.22 Å), B:Gln166 (3.79 Å). Overall, *in silico* docking study revealed that the three best compounds from *S. album* exhibited better inhibitory activity than positive control ciprofloxacin. This finding suggests that the binding interactions may serve as a potential mechanism accountable for the inhibition of *S. aureus* associated skin cancer.

### 3.2 Molecular dynamics

**FIGURE 5:**
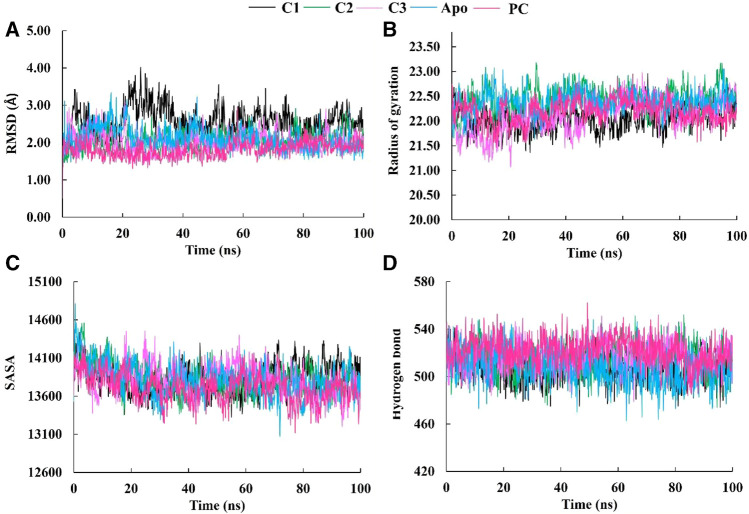
The molecular dynamics simulation study of SarA protein of *Staphylococcus aureus* and C1, C2 and C3 complexes compared to the apo protein and positive control (PC) ciprofloxacin; root mean square deviation **(A)**, radius of gyration **(B)**, solvent accessible surface area **(C)**, and hydrogen bond **(D)**.

Molecular dynamics (MD) simulations are performed on protein-ligand complexes to gain a detailed understanding of their dynamic behavior at an atomic level (Shukla and Tripathi, 2020). Which, enables a dynamic view of their behavior, providing valuable information for drug discovery, understanding biological processes, and elucidating structure-function relationships (Guterres and Im, 2020). In this molecular dynamics simulation, the three best docked complexes was performed to validate the docking conformational stability and its rigidity at 100 ns time-dependent manner which enables the uncovering of potent inhibitors. The root means square deviations (RMSD), solvent accessible surface area (SASA), the radius of gyration (Rg), and the hydrogen bonds of the SarA protein of Staphylococcus *aureus* and the best docked ligand complex are shown in Figure 5.

The Root Mean Square Deviation (RMSD) is a widely used metric for evaluating structural variations and determining the stability, precision, and conformational changes in protein-ligand complexes (Singh et al., 2023). In this study, we conducted an analysis of the RMSD values for the complex formed between drug candidate compounds and the SarA protein over a simulation period of 100 ns. The average RMSD value for complexes C1, C2, C3, Apo and PC was 2.57 Å, 2.07 Å, 2.03 Å, 2.10 Å, and 1.18 Å respectively (Table 3). According to the simulation result, the C1 complex showed the highest RMSD value between 20 and 40 ns which was 2.5 Å to 4.0 Å and exhibited increased RMSD value after 20 ns by following the upward movements. This might be due to the conformational variability of C1 complex after 20 ns. On the other hand, other two complexes C2 and C3 maintained a stable RMSD value from the very beginning to the rest of the simulation period which was almost similar to the RMSD profile of Apo protein and positive control (PC) ciprofloxacin (Figure 5A). The results of the analysis indicate that complexes C2 and C3 exhibited higher structural stability and maintained a consistent conformation throughout the simulation period in comparison to the C1 complex. The lower and more stable RMSD values observed for C2 and C3 suggest that they performed better in terms of overall structural integrity and robustness. Based on the RMSD findings, it can be concluded that C2 and C3 possess enhanced stability compared to the C1 complex.

**TABLE 3:** The average values of MD parameters RMSD, Rg, SASA and RMSF for C1, C2, C3, and PC (Positive control, ciprofloxacin).

Parameters	C1	C2	C3	Apo	PC
RMSD	2.57	2.07	2.03	2.10	1.81
Rg	22.05	22.43	22.10	22.38	22.19
SASA	13826.88	13824.28	13832.02	13810.52	13712.75
RMSF	1.62	1.42	1.58	1.76	1.42

The radius of gyration (Rg) is a crucial metric that provides insights into the overall size of a protein-ligand complex (Miraz et al., 2023). It is a fundamental measure used to assess the structural fluctuations occurring during molecular dynamics (MD) simulations. By analyzing Rg, we can quantitatively evaluate the degree to which the protein’s structure changes the simulation (Singh et al., 2021). The average Rg values for each complexes ranging from 22.05 to 22.43 (Table 3). The Rg profile of the three complexes showed a similar trend with the Apo protein and positive control throughout the whole simulation period ranging from 21 to 23 Å (Figure 5B). A lower Rg value is commonly associated with a compact and rigid structure (Islam et al., 2021). This suggests that all complexes are positioned relatively closer to their center of mass, indicating a folded or globular conformation, and did not change throughout the 100 ns simulation period.

The Solvent Accessible Surface Area (SASA) analysis is employed to determine the surface area of a molecule that is accessible to solvent molecules that is accessible to solvent molecules, can provide insights into the stability and folding of proteins (Baruah et al., 2022). The average SASA values for each complexes ranging from 13712.75 to 13832.02 (Table 3). In this study, the SASA profile of three complexes showed decreasing trend from the beginning of the simulation and after 20 ns had a stable profile that was almost similar to the Apo protein and ciprofloxacin (PC) (Figure 5C). It suggests that the complexes, Apo protein and ciprofloxacin share similar levels of accessibility to the surrounding solvent environment. This similarity in SASA values may imply similar levels of flexibility, exposure to functional sites, or potential for interactions. This implies that the ligand-binding regions in all complexes likely serve comparable roles or functions.

The analysis of hydrogen bonds in molecular dynamics (MD) simulations of protein-ligand complexes is crucial for understanding the nature and stability of their interactions (Takano et al., 2022). Hydrogen bonds play a vital role in determining the specificity and strength of binding between the protein and ligand (Wohlert et al., 2022). By monitoring hydrogen bond formation and breaking events during the simulation, one can gain insights into the dynamics of the complex, including transient interactions and the stability of the binding interface. The hydrogen bonds of C1, C2, and C3 complexes were similar to Apo protein and positive control (PC) ciprofloxacin, and did not change during the 100 ns simulation period (Figure 5D). These similar hydrogen bonds of all complexes suggest that the binding interactions between the protein and ligand are consistent and maintained throughout the simulation. It indicates that specific amino acid residues in the protein form stable hydrogen bonds with the ligand over time, contributing to the overall stability of the complex.

**FIGURE 6:**
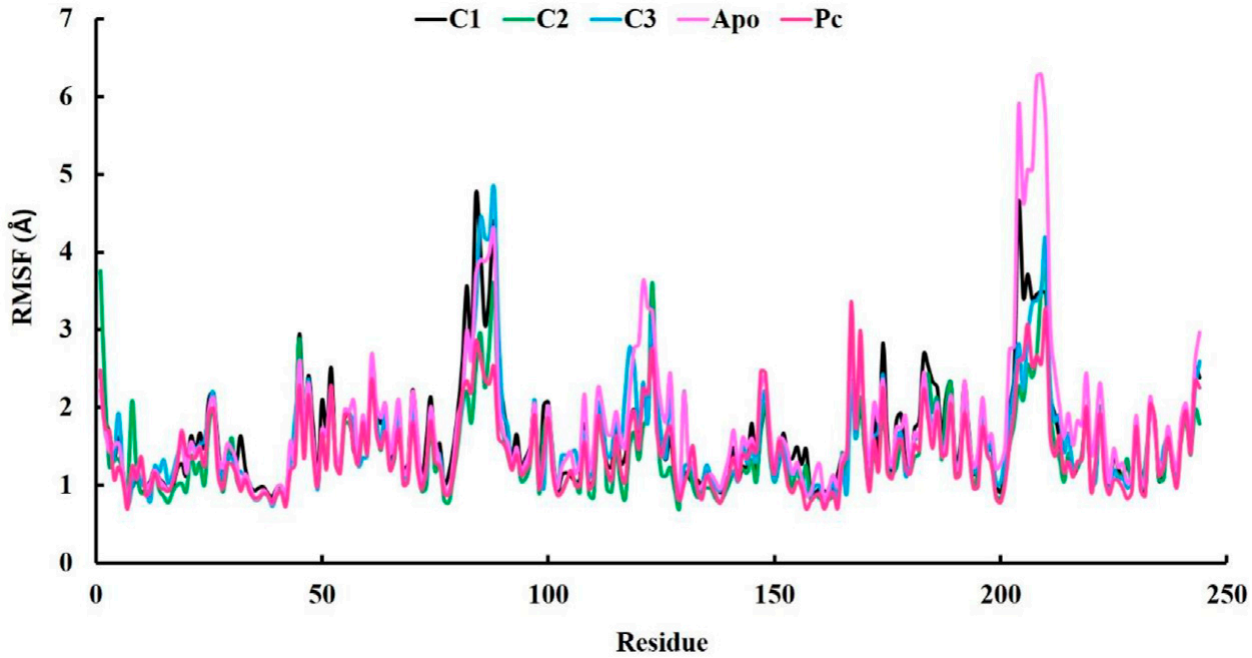
The analysis of Root-mean-square fluctuation (RMSF) at 100 ns molecular dynamics simulations periods.

In our research, we also employed RMSF (Root Mean Square Fluctuation) analysis to investigate how individual atoms or residues in a biomolecular system behave in terms of flexibility and dynamics. This analysis also helped us pinpoint specific residues responsible for these fluctuations (Akash et al., 2023b). Accroding to the RMSF results, the average values ranging from 1.42 to 1.67 Å which is outstanding results (Table 3). Among the top three compounds we studied, their RMSF profiles closely resembled that of the positive control, ciprofloxacin, as well as the Apo protein. However, we observed that certain residues exhibited significant fluctuations, exceeding 4 Å, specifically residues numbered 85–89 and 204–210 (Figure 6). Despite the overall similarity in the RMSF profiles of these compounds, some residues within the complexes displayed a higher degree of flexibility and mobility during our simulations. This increased flexibility in these particular residues may have important functional implications, such as aiding in binding events, accommodating structural changes, or playing a role in molecular recognition processes.

### 3.3 MMPBSA binding energy

**FIGURE 7:**
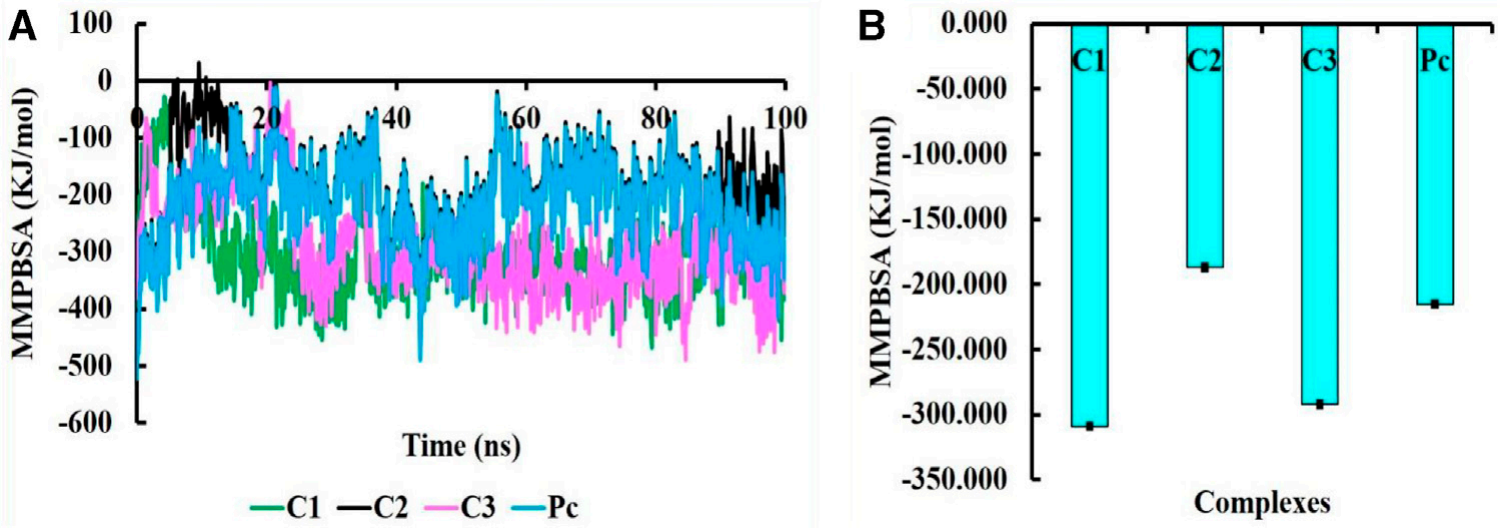
The MMPBSA binding free energy of C1, C2, C3, and PC complexes, where MMPBSA energy at 100 ns simulation period **(A)** and average MMPBSA binding free energy **(B)**.

The MMPBSA binding energy represents the net energy change associated with the formation of a protein-ligand complex compared to the unbound components. In our study, the binding free energy of protein-ligand interactions was determined using Molecular Mechanics/Poisson-Boltzmann Surface Area (MMPBSA) calculations. The MMPBSA binding free energies of all complexes were evaluated at a simulation period of 100 ns, as depicted in Figure 7. The average MMPBSA binding free energies for complexes C1, C2, C3 and PC were found to be −309.08 ± 1.92, −186.90 ± 2.40, −292.07 ± 2.02, and −215.193 ± 1.81 kJ/mol, respectively, herein it has been shown that the MMPBSA binding energy for C1 and C2 were better that the positive control (Table 4). This negative MMPBSA binding energy indicates a favorable binding interaction, indicating that the binding of the ligand to the protein is energetically favorable and stable.

**TABLE 4:** The average MMPBSA binding energy with other energies terms for each complexes.

Parameter	C1	C2	C3	PC
EpotRecept+	−7639.32821	−7429.266547	−6733.317016	−6716.32
EsolvRecept+	−32554.71565	−32994.28697	−33843.83064	−33813.8
EpotLigand+	−131.0693027	−131.5396274	−103.8731738	−98.8732
EsolvLigand-	−480.7263047	−470.9471429	−562.047973	−532.048
EpotComplex-	−8076.981531	−7718.179175	−7109.620093	−7119.62
EsolvComplex	−32422.93294	−33120.60407	−33845.08681	−33825.1
MMPBSA (mean ± SE)	−309.083 ± 1.92	−186.904 ± 2.40	−292.080 ± 2.02	−215.193 ± 1.81

**FIGURE 8:**
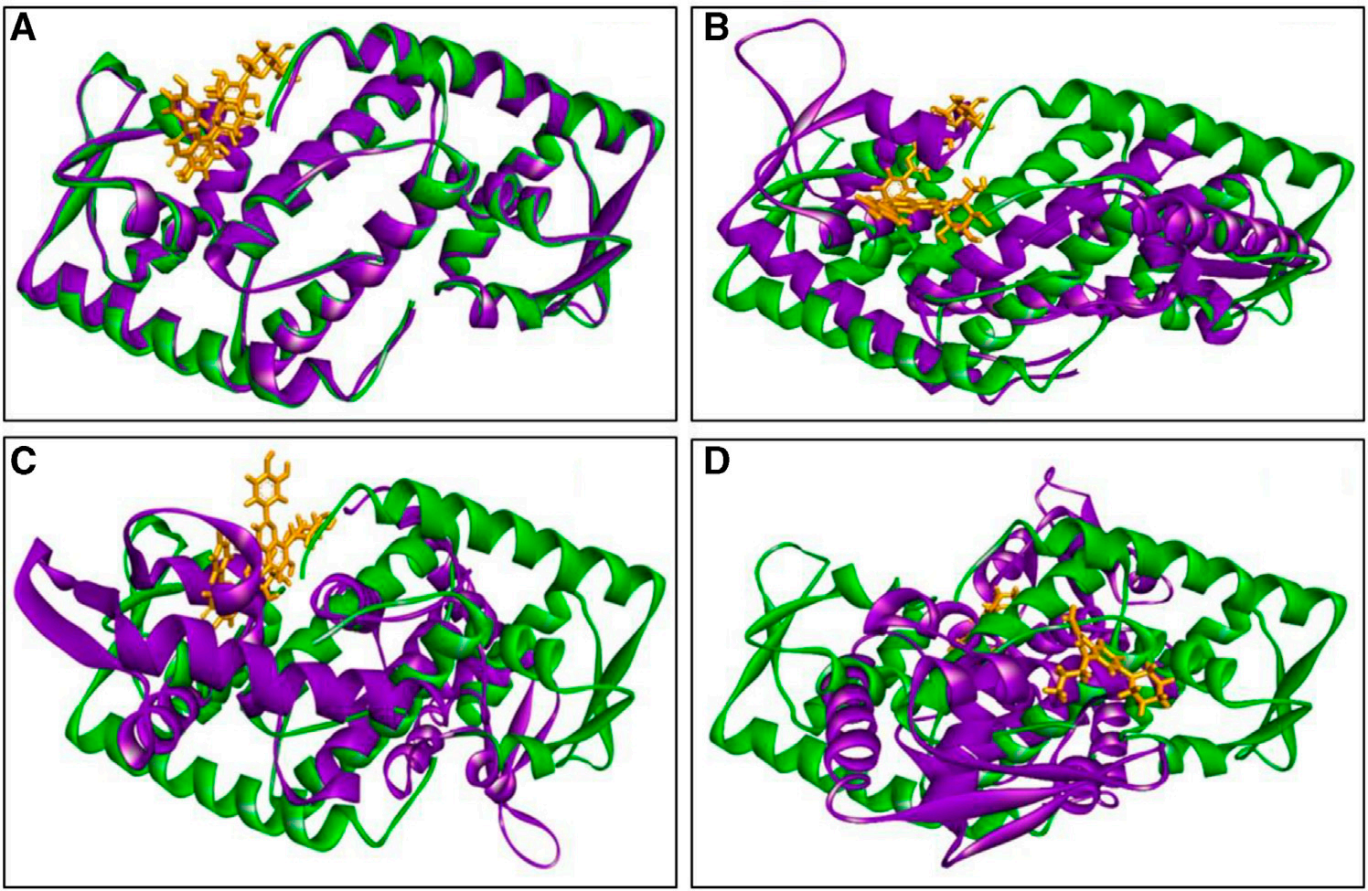
The superimposition of both docked (green colour) and simulated (at 100 ns, violet colour) complexes. Where **(A)** C1, **(B)** C2 **(C)** C3, and **(D)** PC, and ligand defined as gray colour. Figures were generated by using Discovery Studio v21.1.0.0.

The inclusion of structural information, both in the docked and simulated states is shown in Figure 8. The superimposition revelaed that all three lead compounds exhibited strong binding activity at the binding pocket whereas both docked and simulated complexes showed almost similar structure compared to the positive control.

**FIGURE 9:**
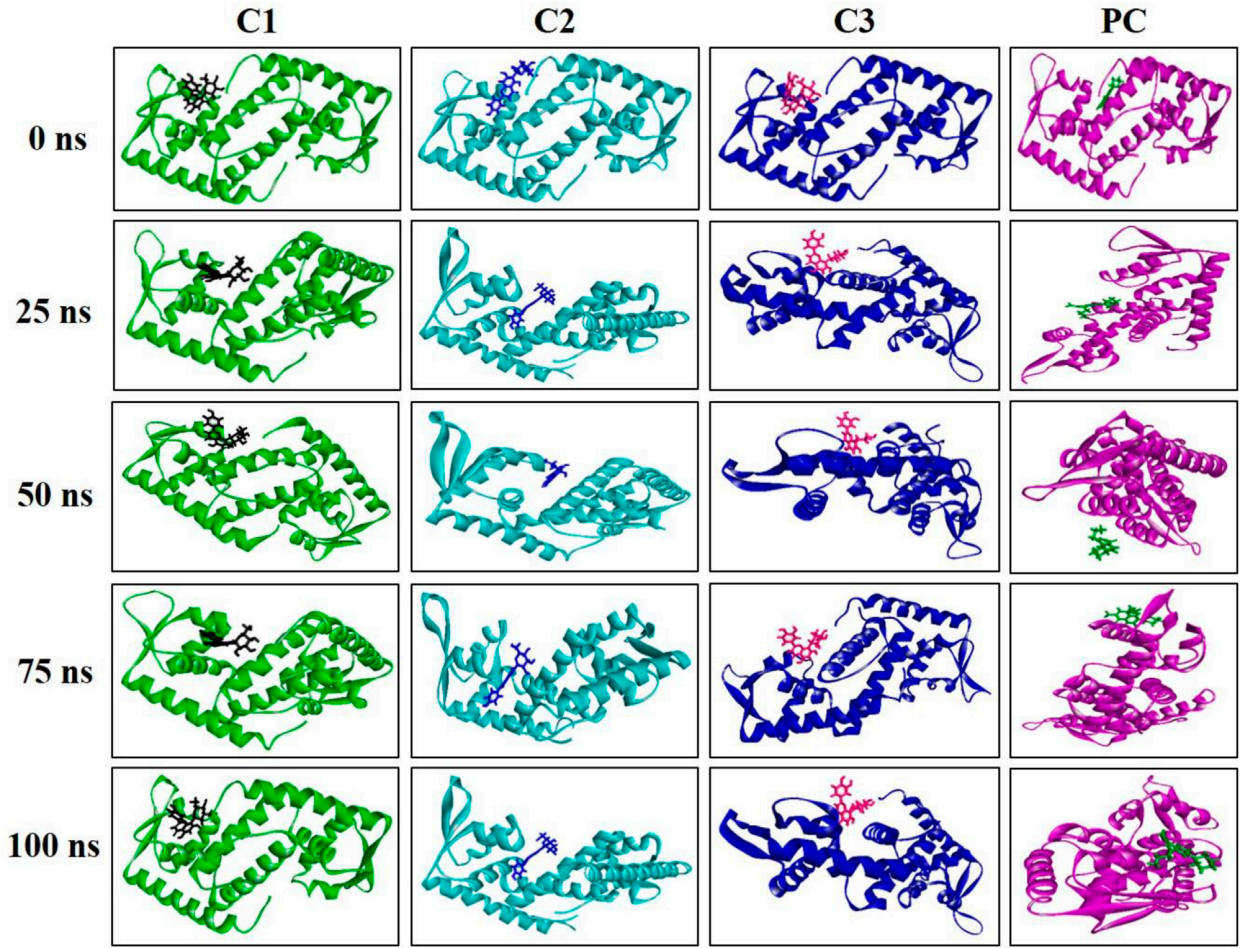
Snapshot of C1, C2, C3, and PC complexes at 0, 25, 50, 75, and 100 ns simulation perioid. Figures were generated by using Discovery Studio v21.1.0.0.

The MD simulation snapshot of complexes C1, C2, C3 and PC at 0, 25, 50, 75, and 100 ns are shown in Figure 9. According to this results, both C1 nad C2 compounds remain sustain at the binding pocket of the target protein 2fnp throughout the simulation period where significant structural change were not observed for this both complexes (Figure 9). Similarly, C3 compounds also almost suatain with the target protein however there was slight structural changes of the protein was observed particularly at 75, and 100 ns. On the other hand, the positive control drug was splited out from the target protein at 50 ns however at rest of the time control drug was bounded with target protein. Overall, superimposion results suggest that all three compounds were strongly bounded with the target protein throughout the simulation period compared to the positive control.

### 3.4 Principle components analysis (PCA)

PCA is commonly used in MD simulations to analyze and understand the conformational dynamics and structural variations of apo form and protein-ligand complexes (Paris et al., 2014; Zarezade et al., 2018; Kitao, 2022). It was conducted to gain insights into the alterations in conformational dynamics that occur upon the binding of the ligand. PCA helps uncover the most significant structural changes and collective motions occurring in the protein-ligand complex during a molecular dynamics (MD) simulation (Kaur Bijral et al., 2022). In our study, PCA was utilized to explore the overall variability among protein-ligand complexes, including comparisons with the apo form. The techniques employed included the diagonalization of covariance matrices and mathematical eigenvalues, which provided information about the magnitude and direction of structural fluctuations. Furthermore, the eigenvectors represented the directions of these fluctuations (Alom et al., 2023).

**FIGURE 10:**
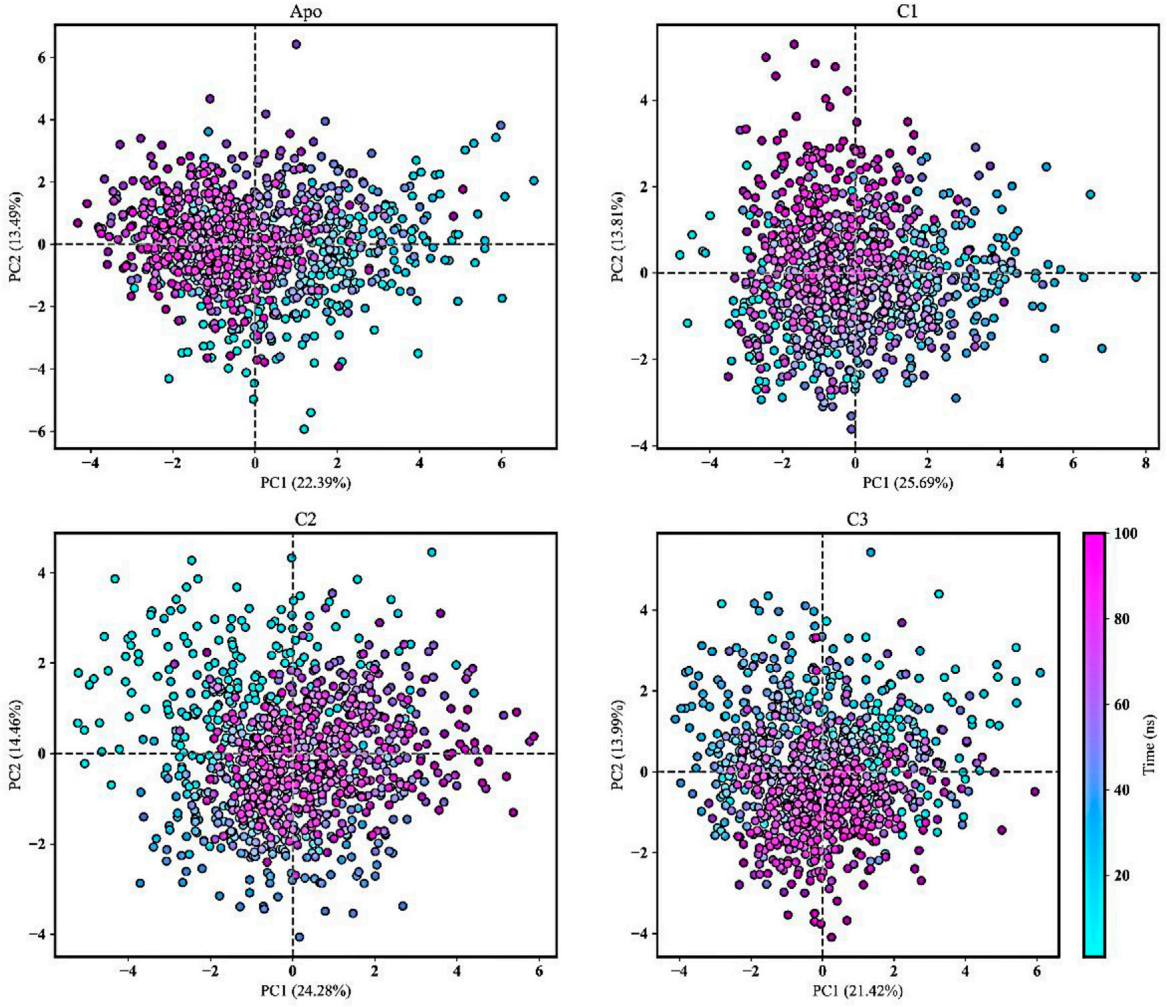
Principal component analysis (PCA) was conducted to analyze the movements of the Apo form and protein-ligand complexes (C1, C2, and C3) over a 100 ns molecular dynamics simulation. The PCA trajectories transitioned from cyan to purple as the simulation runtime progressed. The first and second principal components were plotted and the simulation time was presented as color map.

Figure 10 showcased PCA results derived from all the trajectories incorporating both coordinate information and pertinent structural features. The total explained variance ratio of Apo, C1, C2, and C3 was 35.89%, 39.51%, 38.74%, and 35.42% correspondingly. The conformational distributions of the apo form appear to be similar to those of the protein-ligand complexes. The results of the PCA analysis indicated that there is a widespread distribution of the apo form and protein-ligand complexes, suggesting that they exhibit conformational stability throughout the trajectory. Additionally, the distribution of the protein-ligand complexes closely resembled that of the Apo form, indicating a similarity in their overall structural characteristics. Moreover, the complexes did not display substantial structural variations throughout the MD simulation. The presence of low variance eigenvectors further confirmed the stability of the conformational states, suggesting that despite their dynamic nature, the complexes maintained consistent structural configurations.

### 3.5 ADMET analysis

ADMET (absorption, distribution, metabolism, excretion, and toxicity) analysis was performed for top three ligands to find out a lead compound. We found that CID: 5280441 (C1), CID: 162350 (C2) and CID: 5281675 (C3) followed Lipinski’s rule although having few violations. The bioavailability score of C1, C2, and C3 compounds was 0.55, 0.55, and 0.17 respectively, which indicates that these compounds were physiologically active as the bioavailability score of a compound determines its physiological activity (Hosen et al., 2023). According to the solubility scale, insoluble < −10 < poorly < −6 < moderately < −4 < soluble < −2 < very <0 < highly water soluble (Daina et al., 2014), the water solubility of compounds C1 (−2.845), C2 (−2.812) and C3 (−2.905) was found to be water-soluble. Compounds C2 showed the highest human intestinal absorption rate of 64.729% followed by C1 (46.695%) and C2 (43.733%). The Blood brain barrier and CNS permeability of all three compounds exhibited negative results (Table 5), indicaing that compounds are less likely to cross the BBB and less likely to permeate the central nervous system (CNS) as well (Shaker et al., 2023). Moreover, three screened compounds showed positive results in the human ether-a-go-go (hERG) l inhibitor test, and no toxicity was found in hepatotoxicity and AMES tests (Table 5). It suggests that these three compounds are suitable for further lab experiments. So, these compounds could be used as lead compounds to develop a drug against antibiotic resistant *S. aureus*.

**TABLE 5:** Pharmacological and toxicity prediction of the screened compounds of *S. album* from SwissADME and PKCSM tools where every compound had almost favorable drug-likeness properties.

Parameter	CID: 5280441	CID: 162350	CID: 5281675
Molecular weight	432.38	432.38	448.38
Molecular formula	C_21_H_20_O_10_	C_21_H_20_O_10_	C_21_H_20_O_11_
Hydrogen bond donor	7	7	8
Hydrogen bond acceptor	10	10	11
Rotatable bonds	3	3	3
LogP	0.0917	0.0917	−0.2027
Surface Area	173.994	173.994	178.788
Bioavailability score	0.55	0.55	0.17
Water solubility	−2.845	−2.812	−2.905
Human intestinal absorption	46.695	64.729	43.733
Blood brain barrier	−1.449	−1.375	−1.639
CNS permeability	−3.834	−3.754	−4.018
P-Glycoprotein 1 inhibitor	No	No	No
CaCo2 Permeability	−0.956	−0.618	−1.25
CYP2D6 substrate	No	No	No
Oral Rat Acute Toxicity (LD50)	2.595	2.558	2.572
AMES Toxicity	No	No	No
Hepatotoxicity	No	No	No
hERG 1 Inhibitor	No	No	No

### 3.6 Pharmacophore mapping

**FIGURE 11:**
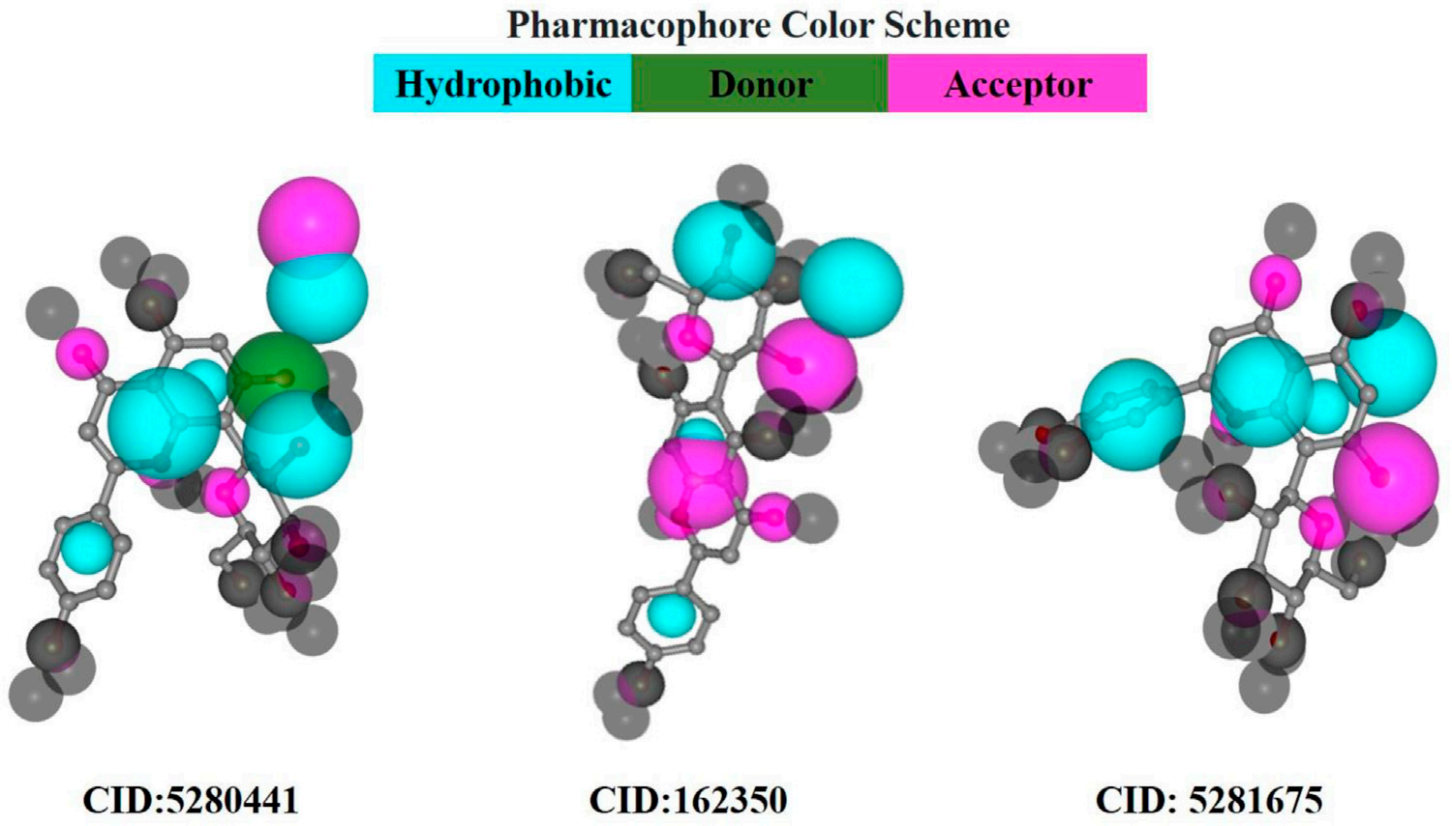
Pharmacophore model of three best-docked compounds carried out by PharmMapper online server (https://www.lilab-ecust.cn/pharmmapper/).

In pharmacophore mapping, the fit score is a quantitative measure used to assess the degree of similarity or fit between a pharmacophore model and a given molecular structure or compound (Opo et al., 2022). Herein, we analyzed the pharmacophore mapping of all three compounds for SarA protein (Figure 11), and the fit scores of compounds C1 and C3 are 4.51 and 3.701, respectively, which are higher than that of C2 with 2.95 (Table 6). These higher fit scores of the compounds indicate a closer match between the pharmacophore model and the compounds, suggesting a higher likelihood of the compounds exhibiting similar biological activity to the target or reference molecule. On the other hand, the normalized fit score provides a standardized measure of the similarity between a compound and a pharmacophore model, enabling comparisons across different models and compounds in pharmacophore mapping studies (Thangavel and Albratty, 2022; Tyagi et al., 2022).

**TABLE 6:** The results of pharmacophore mapping analysis of three compounds for SarA protein.

Parameters	CID: 5280441 (C1)	CID: 162350 (C2)	CID: 5281675 (C3)
Fit score	4.51	2.95	3.701
Normalized fit score	0.7375	0.6443	0.7403
Hydrophobic centre	3	2	3
Positively charged centre	0	0	0
Negatively charged centre	0	0	0
H bond donor	1	0	0
H bond acceptor	1	2	1
Aromatic ring	0	0	0

In our study, we revealed the highest normalized fit scores for compounds C1 and C3, with 0.7375 and 0.7403, respectively, followed by C2 with 0.6443 (Table 6). The normalized fit score of a compound is usually between 0 and 1, where 1 represents a perfect match between the compound and the pharmacophore model. This finding suggests that C2 and C3 were almost similar, and both compounds match the pharmacophore model well. Additionally, compounds C1 and C3 generated similar hydrophobic centers and one hydrogen bond acceptor each. On the other hand, C2 exhibited two hydrophobic centers with one hydrogen bond acceptor. Interestingly, except for C1, none of the other compounds exhibited a hydrogen bond donor. Moreover, all compounds C1, C2, and C3 did not generate any positively charged centers, negatively charged centers, or aromatic rings (Table 6).

### 3.7 *In vitro* antibacterial activity

**FIGURE 12:**
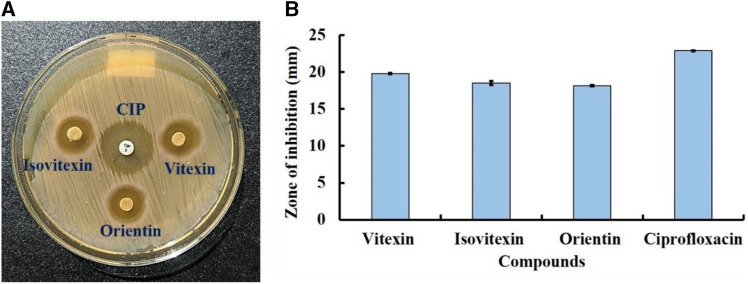
Antibacterial activity of compounds Vitexin (CID:5280441), Isovitexin (CID:162350), and Orientin (CID:5281675) against skin cancer associated S. aureus at 50 µg/disc concentration. Where, **(A)** clear zone in disc diffusion method and **(B)** zone of inhibition. Ciprofloxacin (CIP) used as a positive control.

To validate the findings of our *in silico* analysis, we conducted an *in vitro* assessment of the antibacterial activity of three key compounds, namely, Vitexin (CID:5280441), Isovitexin (CID:162350), and Orientin (CID:5281675) from *S. album* against *S. aureus* is shown in Figure 12. The result revealed that all compounds exhibited almost similar level antibacterial activity. The compounds Vitexin showed the strong activity with zone of inhibition 19.8 ± 0.11 mm followed by Isovitexin and Orientin with zone of inhibition 18.53 ± 0.27 mm and 18.16 ± 0.08 mm respectively compared to the positive control ciprofloxacin with 22.90 ± 0.05 mm. On the other hand, negative solvent control doesnot effect on the growth of *S. aureus* (Supplementary Figure S1). Hence, based on the comprehensive *in vitro* investigation, it can be inferred that these three compounds possess the potential to inhibit *S. aureus* associated with skin cancer. Nonetheless, further molecular studies are imperative to provide additional confirmation and insight into their mechanisms of action.

## 4 Conclusion

In this research, we find out the compounds CID: 5280441, CID: 162350 and CID: 5281675 from *S. album* showed strong inhibitory activity against the SarA protein of *S. aureus* through molecular docking study. This was further confirmed by molecular dynamic simulation and MMPBSA binding free energy at 100 ns timeframe. Moreover, all of these compounds followed the drug candidate criteria through ADMET and pharmacophore model studies. *In vitro* antibacterial activity of this three lead compounds, namely, Vitexin, Isovitexin, and Orientin exhibited strong inhibitory activity of skin cancer associated *S. aureus*. Therefore, these three compounds could be used as promising compounds with antibacterial activity against for *S. aureus* associated skin cancer in humans. However, more experimental studies and modification of ligands with different functional groups need to be carried out for further confirmation and improvement of inhibitory activity.

## Data Availability

Data are available from the corresponding author upon reasonable request.
