# Supplementary material for: Mechanistic insight of Staphylococcus aureus associated skin cancer in humans by Santalum album derived phytochemicals: an extensive computational and experimental approaches
Source: Front Chem. 2023 Nov 21;11:1273408. doi: 10.3389/fchem.2023.1273408 (PMC10702530; doi:10.3389/fchem.2023.1273408)
Supplement: Supplementary file 1 [file DataSheet1.PDF]

Supplementary file

Mechanistic insight of *Staphylococcus aureus* associated skin cancer in humans by *Santalum album* derived phytochemicals: An extensive computational approach

S1. Details procedure of YASARA

Molecular dynamics (MD) simulation is a computational technique that allows you to study the behavior of molecules in motion. Here are the steps to perform MD simulation using YASARA software:

1. Launch YASARA and load the structure of our system of interest. You can load a structure from a file, from the PDB database, or build it from scratch using YASARA's modeling tools.
2. Prepare our system for simulation by adding hydrogens, assigning charges, solvating, and adding counter-ions. You can use the menu option Analyze > Prepare macro-molecule for simulation to automate this process.
3. Choose the force field and simulation parameters (provided in manuscript) that suit our system and research question. YASARA supports several force fields, such as AMBER, CHARMM, and YAMBER however we use AMBER. You can also choose the simulation cell shape, temperature, pressure, time step, and integration algorithm. You can use the menu option Analyze > Run simulation to access these options.
4. Run the simulation and monitor its progress. You can use the menu option Analyze > Simulation status to see various statistics and plots of the simulation, such as potential energy, temperature, pressure, RMSD, etc. You can also visualize the trajectory in real time using YASARA's molecular graphics features.
5. Analyze the results of the simulation and generate a report. You can use the menu option Analyze > Analyze trajectory to perform various analyses on the trajectory, such as secondary structure, hydrogen bonds, contacts, RMSF, DCCM, etc. YASARA will automatically create a detailed scientific report with plots and tables ready for publication. You can also use your own custom scripts or plugins to perform additional analyses.

For more details, please visit

http://www.yasara.org/mdsetup.htm

http://www.yasara.org/mdanalysis.htm

S1. The procedure for calculating Principal Component Analysis (PCA) from the provided Python code is as follows:

Data Conversion: The reviewer mentions that a .sec file generated by Yasara's md_analyze was converted to GROMACS' .xtc format. This .xtc file contains snapshots of the system's structural data over a 100 nanosecond (ns) simulation.

Python Code: The PCA analysis was performed using the Python code provided. Below is a step-by-step breakdown of the code:

```
import numpy as np

import pandas as pd

import matplotlib.pyplot as plt

from sklearn.decomposition import PCA

import MDAnalysis as mda

import nglview as ng

from sklearn.preprocessing import StandardScaler

from sklearn.decomposition import PCA
```

The necessary Python libraries, including NumPy, pandas, Matplotlib, scikit-learn (for PCA), MDAnalysis (for loading trajectory data), and NGLView (for molecular visualization), are imported.

```
# Load trajectory and topology

u = mda.Universe("2fnp_16235000000.pdb", "2fnp_162350.xtc")
```

The code loads the molecular dynamics trajectory from the .xtc file and its corresponding topology from the .pdb file.

```
# Extract all atom coordinates into a 2D array

coordinates = []

for ts in u.trajectory:

    frame_coords = u.atoms.positions

    coordinates.append(frame_coords)
```

It extracts the coordinates of all atoms at each time step in the trajectory and stores them in a 2D array.

```
# Convert to a 2D NumPy array

coordinates = np.array(coordinates)
```

The extracted data is then converted into a 2D NumPy array.

```
# Reshape the data to 2D
```

```
num_frames, num_atoms, num_dimensions = coordinates.shape

coordinates = coordinates.reshape(num_frames, num_atoms * num_dimensions)
```

The data is reshaped from a 3D array to a 2D array, which is a required input for PCA.

```
# Normalize the data

scaler = StandardScaler()

scaled_data = scaler.fit_transform(coordinates)
```

The data is normalized by removing the mean and scaling to unit variance using the StandardScaler from scikit-learn.

```
# Perform PCA

pca = PCA()

pca.fit(scaled_data)
```

Principal Component Analysis (PCA) is performed on the scaled data to extract the principal components.

```
# Scatter plot of the first two principal components

projected_data = pca.transform(scaled_data)

plt.scatter(projected_data[:, 0], projected_data[:, 1])

plt.xlabel('PC1')

plt.ylabel('PC2')

plt.title('C1')

plt.show()
```

Finally, a scatter plot of the first two principal components is generated, providing insight into the structural fluctuations of the system. The plot displays PC1 on the x-axis and PC2 on the y-axis.

Overall, the provided Python code successfully performs PCA analysis on the trajectory data obtained from the .xtc file and visualizes the results through a scatter plot of the first two principal components. This analysis is essential for understanding the structural dynamics and variability of the system under study.

Antibacterial activity

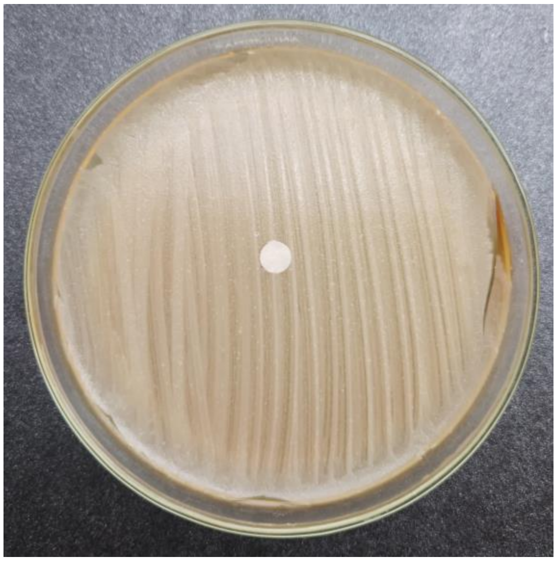

Figure S1. The effect of solvent control (60% methanol) on *S. aureus*.
